# Angioleiomyoma of the Ankle: Case Report and Literature Review of a Rare Benign Soft Tissue Tumor

**DOI:** 10.7759/cureus.55647

**Published:** 2024-03-06

**Authors:** Michael Kozlov, Eyal Levit, Sameera Husain, Edward Mardakhaev

**Affiliations:** 1 Dermatology, Brooklyn College, Brooklyn, USA; 2 Dermatology, Columbia University Irving Medical Center, New York, USA; 3 Dermatopathology, Columbia University Irving Medical Center, New York, USA; 4 Radiology, Montefiore Medical Center, Wakefield Campus, New York, USA

**Keywords:** soft tissue tumor, leiomyosarcoma, imaging, dermatopathology, surgery, pain, ankle, angioleiomyoma

## Abstract

Angioleiomyoma is a benign soft tissue tumor originating in the smooth muscle of blood vessels. It most frequently presents as a painful, free-moving subcutaneous nodule in the lower extremities and is most common in middle-aged women. Angioleiomyoma is rare amongst benign foot neoplasms, and a preoperative diagnosis of angioleiomyoma is rare. We present a case of angioleiomyoma involving the ankle of a 28-year-old female. To prevent patient suffering, we emphasize the importance of an early and accurate diagnosis. Furthermore, we highlight the salient features of angioleiomyoma, which help with the early detection and differentiation of similar malignant variants, including leiomyosarcoma.

## Introduction

Angioleiomyomas are benign subcutaneous tumors typically arising from the smooth muscle of the tunica media [1]. Angioleiomyoma is rare amongst benign foot neoplasms, making up roughly 4.4% of all benign soft tissue tumors and 0.2% of benign foot and ankle lesions [2-4]. The majority of angioleiomyomas are under 2 cm in diameter and are associated with pain in roughly 60% of cases [5,6]. Angioleiomyoma is more common in women than in men, with a male-to-female ratio of 1:1.7, with 67% of cases occurring in patients between 30 and 60 years old with an average age of 47 years [2]. While angioleiomyoma may present anywhere on the body, 67% of cases appear on the lower extremities, but of the cases involving the foot and ankle, only 15.7% are located on the ankle [4]. Since involvement of the ankle is rarely documented, preoperative diagnosis is rare, leading to delays in treatment and prolonged suffering [5]. This is especially important since there are reported cases of malignant transformation, calcification, hemorrhage, hyalinization, and myxoid degeneration associated with angioleiomyoma [7]. 

## Case presentation

A 28-year-old female presented to a podiatrist with a one-year history of pain above the medial malleolus of the left ankle. She described that discomfort initially arose only when wearing shoes or touching the area. This pain worsened over the course of a year and began to occur spontaneously. Upon presentation, her pain was described as sharp at a level of 7/10 on the numeric rating scale, occurred spontaneously several times a day, worsening with palpation, and lasted up to five minutes with radiation up the left aspect of her ankle. No other associated symptoms were present.

Initial clinical examination revealed a small non-fluctuant firm nodule that measured approximately 2-3 mm in size appearing fixed to the skin. No erythema or adjacent swelling were noted. Furthermore, no lymphadenopathy was noted. 

**Figure 1:**
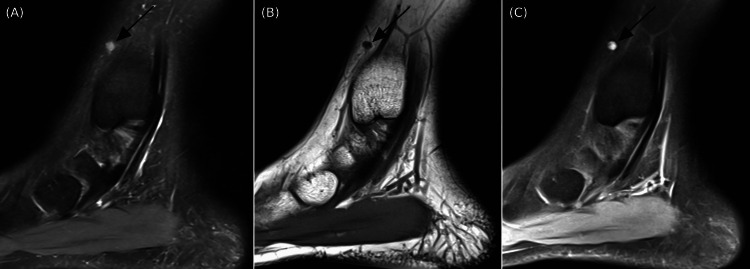
Ankle MRI sagittal images of a small lesion in the subcutaneous fat just below the skin surface at the lateral ankle measuring 3x4x5 mm A: Lesion is hyperintense (black arrow); proton density fat saturation MRI sequence. B: Lesion is hypointense (black arrow); T1-weighted MRI sequence. C: Lesion is avidly enhancing (black arrow); T1-weighted fat saturation MRI sequence with gadolinium contrast.

MRI was requested due to a reported worsening of symptoms in both intensity and frequency. An MRI of her ankle revealed an ovoid 0.5x0.3x0.4 cm enhancing mass in the subcutaneous soft tissues just deep to the skin surface at the level of the distal tibia medially (Figure 1). Impression noted a differential, including neoplasms alongside other etiologies necessitating biopsy.

**Figure 2:**
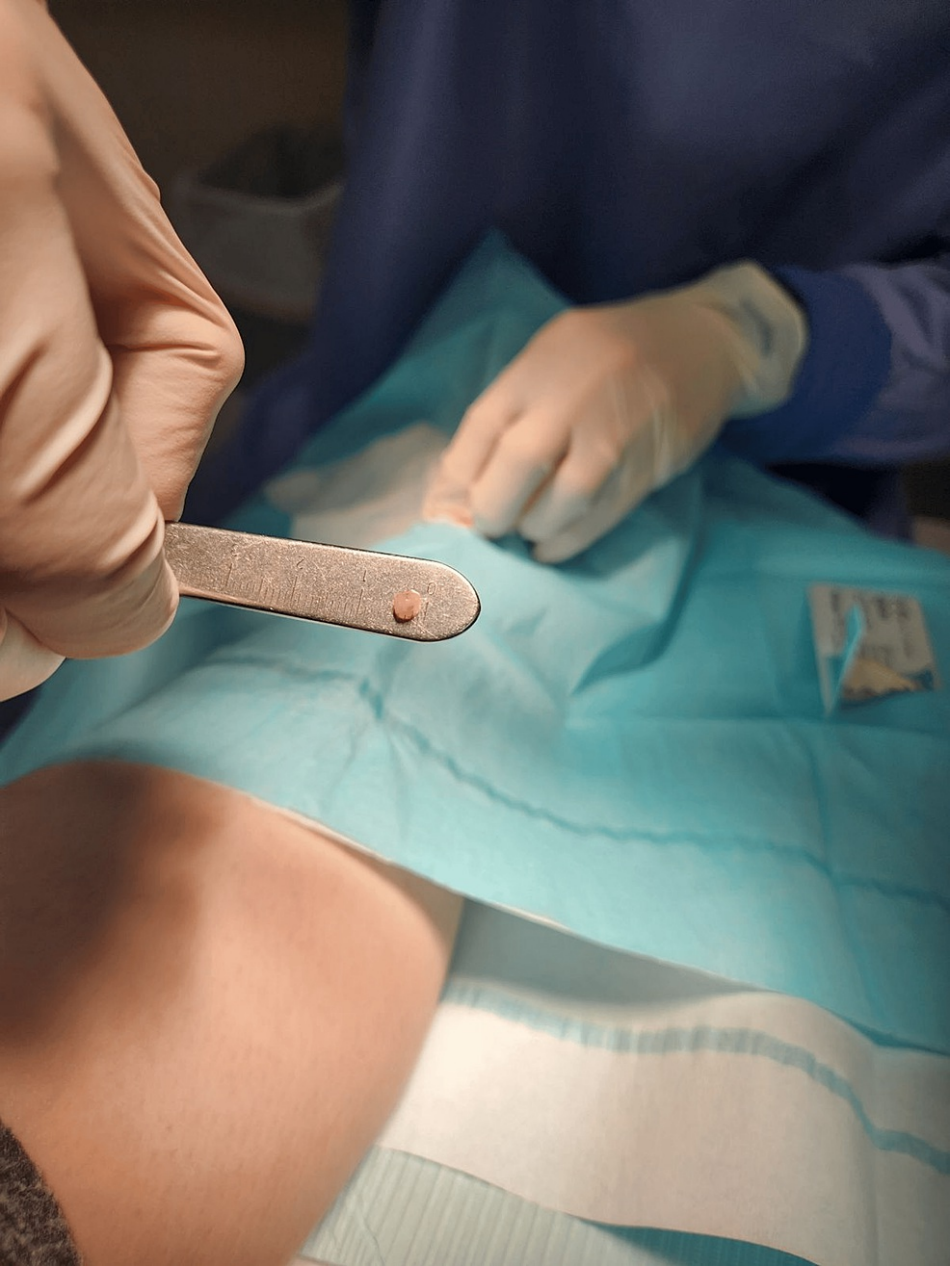
Tissue removed from surgical excision The lesion is round, smooth, and spherical, with a roughly 3 mm diameter.

**Figure 3:**
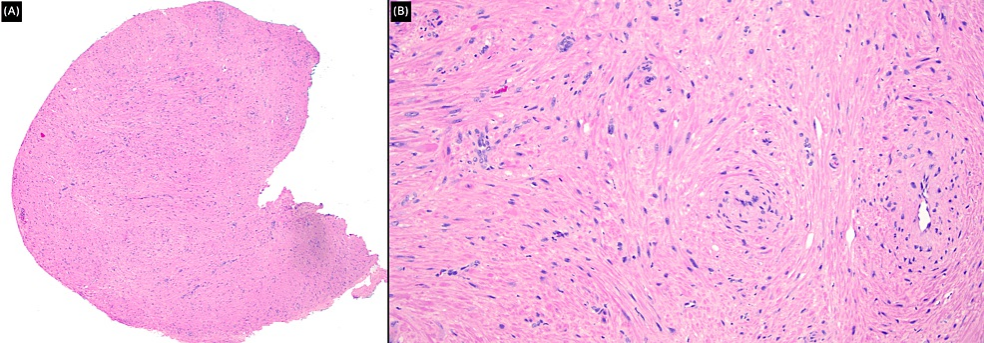
Histopathology examination A: Low-power image showing a circumscribed nodule (H&E stain, magnification x40). B: High-power image exhibiting smooth muscle fibers with straight, blunt-edged nuclei and several capillary-type vascular channels (H&E stain, magnification x200).

We subsequently performed an excisional biopsy (Figure 2). Histopathology examination showed a circumscribed nodule composed of smooth muscle fibers with straight, blunt-edged nuclei and several capillary-type vascular channels, consistent with the solid subtype of angioleiomyoma (Figure 3). The patient reported a complete resolution of symptoms following the excision of the lesion, with a two-year follow-up noting no recurrence of the lesion or her symptoms.

## Discussion

Angioleiomyomas can be designated into three different subtypes: solid, cavernous, and venous, with solid subtypes (67% of cases) outnumbering cavernous (23%) or venous (11%) subtypes [2,4]. These subtypes vary slightly in their typical clinical presentations. Solid subtypes are accompanied by pain in 70% of cases, as opposed to 30% in cavernous cases or 37% in venous cases. Previous literature has attributed the pain associated with angioleiomyoma to the compression of interstitial nerve fibers and ischemia [4]. The pain has been described as burning with electrical aspects and rapidly worsens with light contact or exposure to cold [2]. In the case of our patient, pressure from footwear caused significant pain and occasionally interfered with daily function. A study by Hachisuga et al. also found some subtypes, such as the cavernous subtype, appeared more commonly in men with a male-to-female ratio of 4:1, with a predilection for the head and upper extremities [2]. It should be noted that the venous subtype only slightly favored men and had a roughly even distribution of lower extremity vs upper extremity/head cases. This contrasts with the typical solid presentation that favors females (78% of cases), in whom angioleiomyoma favors the lower extremities (83% of cases). 

The primary method of differentiating these subtypes involves histopathology. The solid subtype of angioleiomyoma is typically composed of smooth muscle cells surrounding vascular channels, which may be capillary-sized but abundant in quantity [2,6]. The cavernous subtype of angioleiomyoma is identified by the dilation of vascular channels and the lowered presence of smooth muscle. Finally, the venous subtype of angioleiomyoma can be characterized by less compact smooth muscle bundles and thickened muscular walls, potentially surrounding the vessels in an organized manner. Some authors note that organizing thrombus, mature fat cells, or lymphocytic infiltrate may be found in some cases of angioleiomyoma [1]. Previous literature also describes cases of hyaline or myxoid degeneration and attributes it to circulatory disturbances, but a clinicopathological reappraisal of cases found that this degeneration didn't frequently accompany painful variants of angioleiomyoma, nor did thrombosis or hemorrhage [1,2]. Although histopathology is diagnostic, immunohistochemistry can be performed in diagnostically difficult cases. Immunohistochemically, angioleiomyomas are generally positive for smooth muscle actin (SMA), HHF35, calponin, caldesmon, and desmin, and vascular markers CD34 and CD31 [8, 9].

While angioleiomyoma has no clear etiology, previous literature has proposed trauma-induced venous stasis, genetic factors, vascular malformations, and hormonal changes with a focus on estrogen as potential factors in the development of angioleiomyoma [4,10]. The differential diagnosis for angioleiomyoma includes ganglionic cyst, giant cell tumor, fibroma, desmoid tumor, tophi, neurofibroma, schwannoma, sarcoma, gouty tophus, glomus tumor, lipoma, hemangioma, foreign-body granuloma, superficial acral fibromyxoma, and leiomyosarcoma.

While histology remains the gold standard in diagnosing angioleiomyoma of the ankle, previous literature has discussed the use of radiography in narrowing down angioleiomyoma in the differential diagnosis [4]. Roughly 30% of all foot and ankle angioleiomyoma cases feature calcifications identifiable by radiography, potentially from minor repetitive traumas. Ultrasonography may show homogeneous structures with well-defined margins, typically showing hypoechogenicity and low-medium vascular density. MRIs detect angioleiomyomas as well defined and bordered by hypointense fibrous capsules. See Table 1 for further information on imaging for angioleiomyoma. It should be noted that histologic examination is necessary to identify whether an angioleiomyoma is solid, cavernous, or venous in nature. 

**Table 1:** Clinical information to help differentiate between angioleiomyoma and leiomyosarcomas

Diagnostic considerations	Angioleiomyoma	Leiomyosarcoma
Clinical Appearance	Typically under 2 cm and rarely over 4 cm. Typically encapsulated [2,10].	Median size of 6 cm with a large range: 0.3-45 cm. Typically not encapsulated [10,15].
Age	The average age is 47 years [2].	The average age is 57 years [15].
Gender distribution	More common in females (roughly 2:1) [2,10].	Roughly even distribution with a slight male preference [16].
Locality	67% in the lower extremity (83% for women) [2].	Disregarding uterine cases, 48% occur in the extremities (more common in the lower extremities), 41% in abdominal or retroperitoneal, and 11% in the trunk [14,15,17].
Recurrence following excision	Less than 1% of cases [2].	Up to 60% of cases [18].
Pain	Up to 70% of cases for the solid subtype [2].	Often presents without pain [10,14].
Imaging	Generally, smaller but small size and non-aggressive lesion features do not exclude sarcoma. Isointense signal to muscle is seen on T1-weighted images and intermediate-high signal on T2-weighted images. T2 hypointense rim may be predictive of angioleiomyoma diagnosis [19].	Areas of internal necrosis or hemorrhage, with masses visible with computed tomography. Isointense signal to the muscle is seen on T1-weighted images and intermediate-high signal on T2-weighted images. Aggressive features, such as surrounding soft tissue invasion, larger size, and peritumoral edema/enhancement [20].
Histology	Three main subtypes [1]: solid, closely compacted smooth muscle with small vascular channels; cavernous, dilated vascular channels and less smooth muscle; venous, thick muscular walls and less compact smooth muscle bundles.	Well-defined intersecting bundles of spindle cells with increased eosinophilic cytoplasm and elongated/hyperchromatic nuclei. Increased mitotic activity may suggest a malignant character [16].

While it is rare, there are recorded cases of angioleiomyoma's malignant evolution to leiomyosarcoma and angioleiomyosarcoma, an aggressive vascular tumor with a five-year survival rate estimated between 20% and 35% [4,11,12]. It should be noted that cases of angioleiomyosarcoma are rare in literature, often not being defined as an independent category of sarcoma, resulting in few reported cases that make generalizations difficult; leiomyosarcoma should be a significant consideration in the differential diagnosis of angioleiomyoma [13]. Leiomyosarcoma makes up about 1% of all soft tissue tumors and 7% of all soft tissue sarcomas [14]. As such, healthcare providers should remain vigilant in treating angioleiomyoma despite its benign nature, even in the absence of pain. Physicians should consult Table 1 to ensure accurate diagnosis of angioleiomyoma. Fortunately, excision is incredibly effective in treating angioleiomyoma, and less than 1% of patients experience recurrence following excision [2].

## Conclusions

We present the case of angioleiomyoma in the ankle of a 28-year-old female. Angioleiomyoma is rare amongst benign foot neoplasms, and our case highlights the importance of interdisciplinary cooperation in achieving an early and accurate diagnosis while minimizing patient suffering. Clinicians should remember that while angioleiomyoma is most common in the lower extremities of middle-aged women, its different subtypes may present anywhere on the body. While angioleiomyoma is benign, clinicians should include aggressive soft tissue tumors such as leiomyosarcomas in their differential diagnosis. As a whole, angioleiomyoma has an excellent prognosis following excision, and our patient reports complete resolution of symptoms following her treatment with no recurrence of the lesion or symptoms.
